# S100A14 as a Potential Biomarker of the Colorectal Serrated Neoplasia Pathway

**DOI:** 10.3390/ijms26157401

**Published:** 2025-07-31

**Authors:** Pierre Adam, Catherine Salée, Florence Quesada Calvo, Arnaud Lavergne, Angela-Maria Merli, Charlotte Massot, Noëlla Blétard, Joan Somja, Dominique Baiwir, Gabriel Mazzucchelli, Carla Coimbra Marques, Philippe Delvenne, Edouard Louis, Marie-Alice Meuwis

**Affiliations:** 1 Laboratory of Translational Gastroenterology, GIGA Institute, University of Liège, 4000 Liège, Belgium; pierre.adam@uliege.be (P.A.); catherine.salee@uliege.be (C.S.); florence.quesadacalvo@hepl.be (F.Q.C.); angela.maria.merli@gmail.com (A.-M.M.); charlotte.massot@chuliege.be (C.M.); edouard.louis@uliege.be (E.L.); 2 GIGA Bioinformatics, GIGA Institute, University of Liège, 4000 Liège, Belgium; arnaud.lavergne@uliege.be; 3 Hepato-Gastroenterology and Digestive Oncology, University Hospital of Liège (CHU), 4000 Liège, Belgium; 4 Pathological Anatomy and Cytology, University Hospital of Liège (CHU), 4000 Liège, Belgiumjoan.somja@chuliege.be (J.S.); p.delvenne@uliege.be (P.D.); 5 GIGA Proteomics, GIGA Institute, University of Liège, 4000 Liège, Belgium; d.baiwir@uliege.be; 6 Laboratory of Mass Spectrometry, University of Liège, 4000 Liège, Belgium; gabriel.mazzucchelli@uliege.be; 7 Abdominal Surgery Department, University Hospital of Liège (CHU), 4000 Liège, Belgium; c.coimbra@chuliege.be

**Keywords:** S100A14, serrated pathway, sessile serrated lesions, proteomics, biomarker

## Abstract

Accounting for 15–30% of colorectal cancer cases, the serrated pathway remains poorly characterized compared to the adenoma–carcinoma sequence. It involves sessile serrated lesions as precursors and is characterized by BRAF mutations (BRAF^V600E^), CpG island hypermethylation, and microsatellite instability (MSI). Using label-free proteomics, we compared normal tissue margins from patients with diverticular disease, sessile serrated lesions, low-grade adenomas, and high-grade adenomas. We identified S100A14 as significantly overexpressed in sessile serrated lesions compared to low-grade adenomas, high-grade adenomas, and normal tissues. This overexpression was confirmed by immunohistochemical scoring in an independent cohort. Gene expression analyses of public datasets showed higher S100A14 expression in BRAF^V600E^-mutated and MSI-H colorectal cancers compared to microsatellite stable BRAF^wt^ tumors. This finding was confirmed by immunohistochemical scoring in an independent colorectal cancer cohort. Furthermore, single-cell RNA sequencing analysis from the Human Colon Cancer Atlas revealed that S100A14 expression in tumor cells positively correlated with the abundance of tumoral CD8^+^ cytotoxic T cells, particularly the CD8^+^ CXCL13^+^ subset, known for its association with a favorable response to immunotherapy. Collectively, our results demonstrate for the first time that S100A14 is a potential biomarker of serrated neoplasia and further suggests its potential role in predicting immunotherapy responses in colorectal cancer.

## 1. Introduction

Colorectal cancer (CRC) is the third most common cancer worldwide and the second cancer when ranked according to the number of deaths recorded in 2018 [1]. The most widely characterized and first described model of carcinogenesis is the “adenoma–carcinoma” sequence, which refers to tumors developing from conventional adenomas. CRC arising from this sequence (70–80% of CRC cases) is often characterized by chromosomal instability and stable microsatellites (MSSs) [2]. In addition to the conventional adenoma–carcinoma sequence, approximately 15–30% of colorectal cancers develop from serrated lesions, representing an alternative tumorigenesis pathway [3]. Tumors arising from the serrated pathway are predominantly located in the right colon and are characterized by somatic mutations in *BRAF* (BRAF^V600E^), hypermethylation of CpG islands, and microsatellite instability (MSI) [4,5].

According to the classification defined in the 5th World Health Organization edition, there are three main categories of serrated lesions: traditional serrated adenoma (TSA), sessile serrated lesion (SSL), and hyperplastic polyp (HP). These subtypes differ in clinical, endoscopic, histopathological, and molecular characteristics [6]. HP corresponds to 70–80% of serrated lesions, rarely leading to adenocarcinoma, unlike SSL and TSA. HP are histologically classified into microvesicular type (MVHP) or goblet cell-rich forms. Satorres hypothesized that an MVHP-SSL-CRC sequence exists, suggesting that MVHP could be SSL precursors [7].

In a large case–control study, it has been demonstrated that patients with SSL alone or conventional adenomas alone have a higher risk of developing CRC than patients without polyps (OR = 2.9 and 2.1, respectively) [8]. Moreover, patients with SSL with dysplasia presented a higher risk of developing CRC (OR = 10.2) than those with advanced adenoma (OR = 3.1). Only 4 to 8% of SSL are detected with dysplasia [9]. However, this proportion is relatively low, considering that 20–30% of CRC arise from the serrated pathway. This discrepancy can be explained by missed endoscopic detection and rapid transformation into CRC. This short transformation time contrasts with the relatively long process of development of colorectal adenomas into CRC [10].

Approximately 75% of SSL with dysplasia display MSI and therefore contribute to the generation of MSI-High (MSI-H) colorectal cancers [11]. MSI-H tumors of familial origin, such as Lynch syndrome, rarely harbor BRAF^V600E^ mutations. It is accepted in the literature that MSI-H CRC present a BRAF^V600E^ mutation derived from serrated lesions, unlike CRC derived from the adenoma–carcinoma sequence, which are MSSs [12]. Indeed, the majority of the serrated pathway CRCs are MSI-H [7]. MSI-H tumors are characterized by a mucinous appearance and prominent infiltration of T lymphocytes within the tumor [13]. Moreover, MSI-H cancers are significantly associated with long-term immunotherapy-related positive responses in colorectal and non-colorectal malignancies [14]. These clinical observations underline the importance of a deeper understanding of the progression mechanisms of SSL and address their comparison to the “adenoma-carcinoma” sequence of transformation.

Very few studies have addressed the characterization of SSL at the protein level [15]; Therefore, the main objective of our study was to identify and validate specific protein markers associated with the serrated pathway by using a label-free proteomic approach. To achieve this, we analyzed protein expression in SSL, low-grade adenomas (LGA), and high-grade adenomas (HGA). We then validated our proteomic findings through immunohistochemistry (IHC) on an independent set of FFPE tissues from different patients (LGA, HGA, HP, SSL, and CRC) and complemented our analysis by exploring public microarray and single-cell transcriptomic datasets.

## 2. Results

### 2.1. S100A14 Is Higher in Serrated Lesions Compared to Adenoma

#### 2.1.1. Proteomic Results

**Figure 1:**
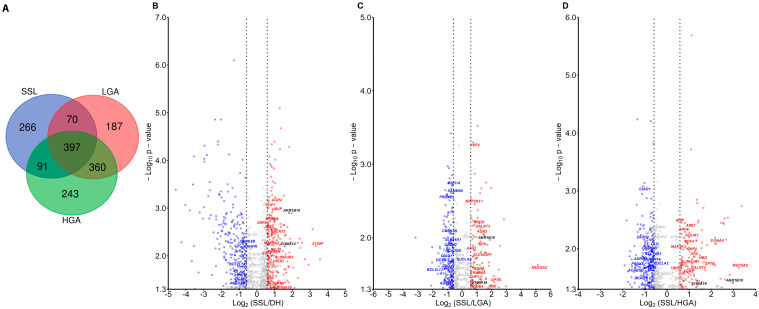
Shotgun proteomic analysis of SSL compared to DH, LGA, and HGA. (**A**) Venn diagram showing the number of proteins found to be statistically significant in comparisons of each lesion type (SSL, LGA, and HGA) versus DH. (**B**) Volcano plots illustrating differentially expressed proteins between SSL and DH, SSL and LGA (**C**), and SSL and HGA (**D**).

Shotgun proteomic analysis identified 6301 proteins across all samples. The Venn diagram shown in Figure 1A highlights the significant protein numbers found for each lesion when compared to adjacent normal tissue [named diverticulitis healthy (DH)] (with 824 significant proteins in SSL, 1014 in LGA, and 1091 in HGA). Figure 1 shows the volcano plot distributions of proteins in each lesion group compared to DH tissues. Supplementary Table S4 provides the results of differential analyses (protein list, gene names, and statistics) and the 6301 protein hits identified within the whole proteomic dataset.

Among the proteins identified and found to discriminate the three lesion groups, S100P was one of the most differentially abundant in SSL compared to DH (Figure 1B). MUC5A was differentially distributed between SSL and conventional adenomas (LGA and HGA) (Figure 1C,D).

S100A14 was more abundant in SSL than in LGA (fold = 2.45, *p* = 0.037), HGA (fold= 2.57, *p* = 0.036), and DH (fold = 2.96, *p* = 0.006) (Dunn’s test). AKR1B10 was more abundant in SSL than in LGA (fold = 2.83, *p* = 0.009), HGA (fold = 7.82, *p* = 0.039), and DH (fold = 3.52, *p* = 0.0013). Due to its consistent differential expression between SSL and adenomas, S100A14 was selected as the main focus for further validation and characterization.

#### 2.1.2. Immunohistochemistry Results

**Figure 2:**
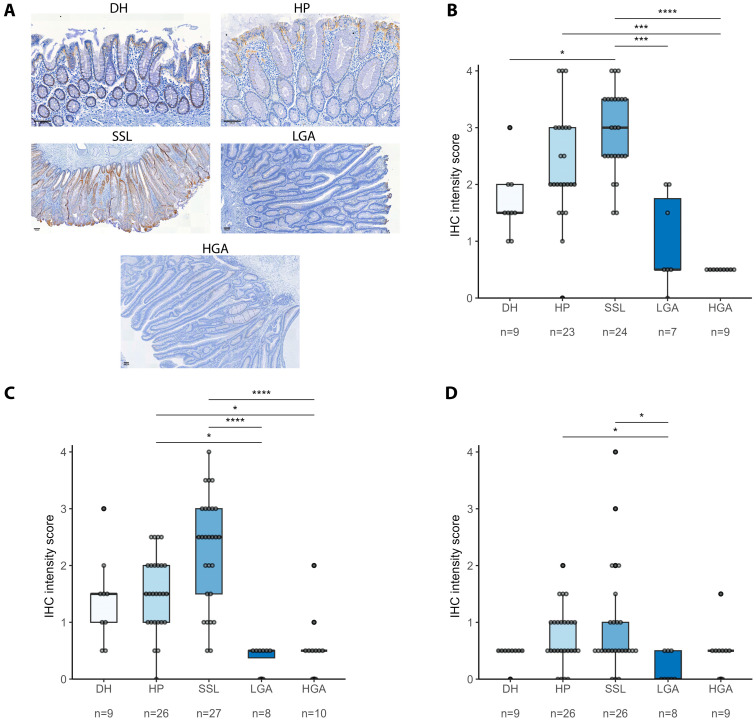
S100A14 IHC expression is higher in serrated lesions compared to adenoma. (**A**) Representative images of S100A14 IHC expression in each group of tissues (**B**–**D**). S100A14 IHC scoring summary in the upper (**B**), middle (**C**), and lower (**D**) crypt portions across all tissue groups and lesion types. *p*-values are represented with * ≤ 0.05, *** ≤ 0.001, and **** ≤ 0.0001 (median ± range).

The S100A14 IHC signal, exclusively cytoplasmic, revealed a S100A14 IHC score distribution confirming its increased expression in SSL compared to other dysplastic lesions (Figure 2). The intensity of staining within middle and upper crypt compartments showed increased intensity in serrated lesions (HP and SSL) compared with DH, LGA, and HGA (Figure 2). A higher S100A14 expression in SSL than in HP was observed, although not reaching significant difference.

In contrast to serrated lesions, S100A14 staining was decreased in LGA and HGA compared to DH. Collectively, proteomic and IHC results showed an increase in S100A14 in serrated lesions compared to adenomas and healthy diverticular margins. AKR1B10 IHC distributions were also increased in SSL compared to those in HGA (Supplementary Figure S1).

### 2.2. S100A14 Is Upregulated in Colorectal Cancers Arising from the Serrated Pathway

**Figure 3:**
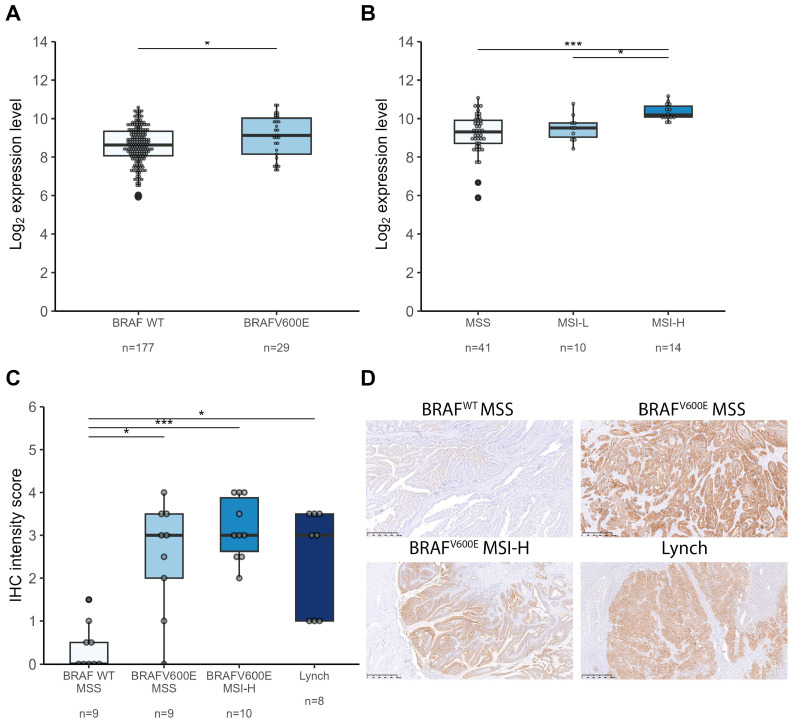
S100A14 is upregulated in colorectal cancers arising from the serrated pathway. (**A**) S100A14 expression levels (log2 expression levels) in colorectal tumors with BRAF^V600E^ mutations (GEO microarray dataset GSE75317) and (**B**) in colorectal tumors grouped based on microsatellite instability status (GEO microarray dataset GSE24550). (**C**) S100A14 IHC expression scoring based on BRAF^V600E^ mutations and MSI/MSS status. (**D**) Representative IHC image of S100A14 staining in each type of tumor. p-values are represented with * ≤ 0.05, and *** ≤ 0.001, (median ± range).

Given that serrated lesions frequently lead to MSI-H CRC harboring BRAF^V600E^ mutations, we investigated S100A14 expression in two public CRC datasets. In the Gene Expression Omnibus (GEO) microarray dataset GSE75317, which includes 177 BRAF^wt^ and 29 BRAF^V600E^-mutated CRC samples, *S100A14* expression was increased in tumors with BRAF^V600E^ mutations (Figure 3A).

The GSE24550 dataset, comprising 41 MSS, 10 MSI-Low (MSI-L), and 14 MSI-H CRC samples, showed tumor *S100A14* expression level distributions based on microsatellite instability status (Figure 3B). *S100A14* expressions were higher in MSI-L and MSI-H compared to MSS tumors, suggesting that higher *S100A14* expression is likely associated with the CRC serrated pathway.

To confirm whether S100A14 is upregulated in cancerous tissues originating from serrated lesions, we analyzed S100A14 IHC scores in FFPE tumors with BRAF^V600E^ mutations and high microsatellite instability (MSI-H), both associated with serrated lesions (Figure 3C,D). Consistent with our findings in SSL and HP, S100A14 staining intensity was significantly higher in MSI-H/BRAF^V600E^ serrated tumors than in sporadic MSS/BRAF WT ones (*p* < 0.001). S100A14 levels were also elevated in MSI-BRAF^WT^ and in MSS-BRAF^V600E^ tissues. No AKR1B10 IHC score significant difference was observed between the groups considered (Supplementary Figure S1).

Taken together, these results show that S100A14 is overexpressed at each stage of the serrated pathway sequence, from serrated lesions to cancer, when compared to normal tissues.

### 2.3. S100A14 Is Associated with Anti-Tumor Immunity

**Figure 4:**
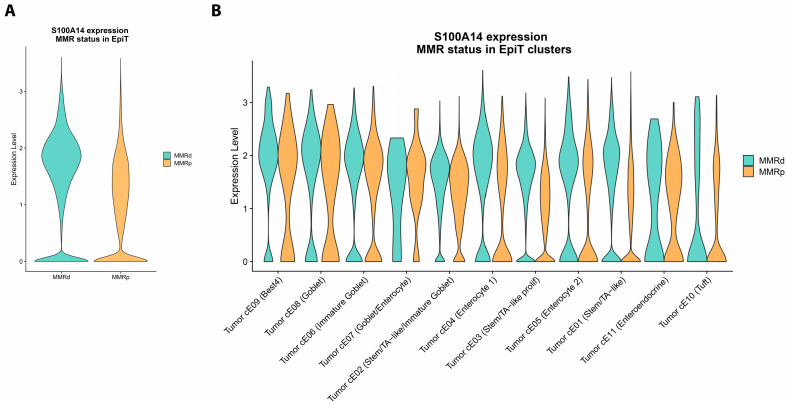
*S100A14* expression is increased in MSI-H CRC cells compared to MSS CRC cells. (**A**) *S100A14* expression in MSI-H colorectal tumors based on single-cell transcriptomic dataset (GSE178341) restricted to the epithelial tumor cell clusters (epiT) with comparison of Mismatch Repair-deficient (MMRd) and MMR-proficient (MMRp) tumors. (**B**) *S100A14* expression within the epiT clustered based on MMR status.

*S100A14* expression in MSI-H CRC was analyzed using a single-cell transcriptomic dataset including 62 CRC donors [16]. *S100A14* was primarily expressed in clusters of tumor cells derived from goblet cells, and an increase in *S100A14* expression was found in MSI-H tumor cells compared to MSS tumor cells (Figure 4A). This upregulation was enhanced in proliferating Stem/TA-like tumor cell clusters (Figure 4B).

**Figure 5:**
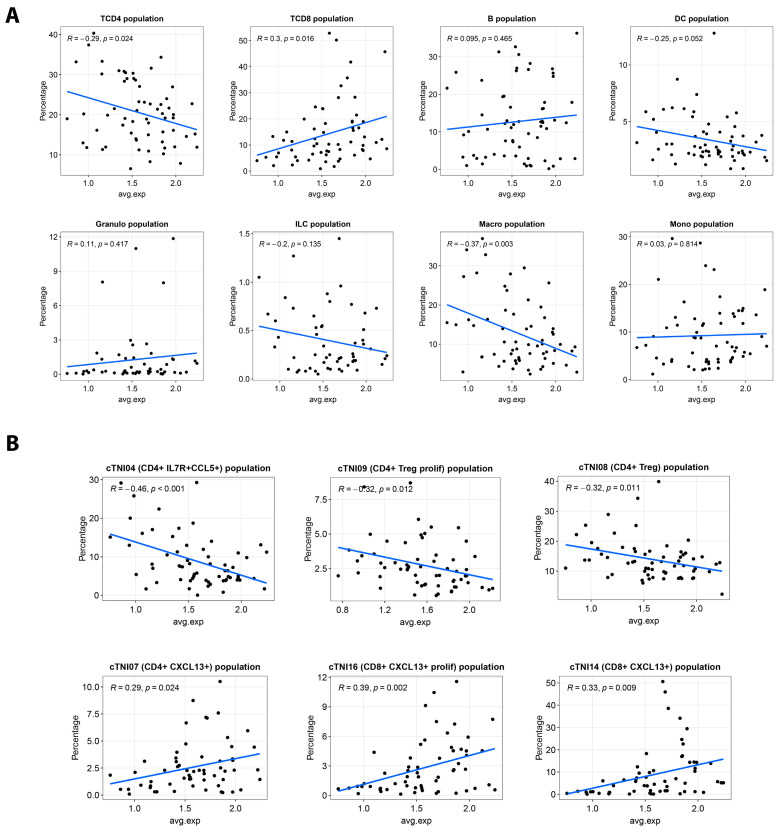
The expression of *S100A14* in epithelial tumor cells is correlated with the intra-tumoral percentage of CD8^+^ cells. Correlations obtained (R, *p*) between the average expression level of *S100A14* in the epithelial tumor cell population of each donor and the proportion of different immune cell populations in the corresponding donor (**A**) or with T cells, NK cells, and ILC subpopulations (**B**) using data from the single-cell transcriptomic GEO dataset GSE178341.

Moreover, *S100A14* expression showed the highest positive correlation with the percentage of CD8^+^ T cells in the tumors (*R =* 0.30*, p =* 0.016), whereas a negative correlation (*R =* −0.29*, p =* 0.024) was observed in the percentage of CD4^+^ T cells and macrophages within tumor samples (Figure 5A). In a more detailed analysis of the T cell populations, we found a significant positive correlation between *S100A14* expression and CD4^+^ CXCL13^+^ (*R =* 0.29*, p =* 0.024), but also with the CD8^+^ CXCL13^+^ T cell population (*R =* 0.33*, p =* 0.009), and more precisely with the CD8^+^ CXCL13^+^ T cell proliferation cluster (*R =* 0.39*, p =* 0.002). Conversely, *S100A14* expression was negatively correlated with CD4^+^ IL7R^+^ CCL5^+^, CD4^+^ IL17^+^, and CD4^+^ Treg cells (Figure 5B). Collectively, these results indicate an association between *S100A14* expression and the cytotoxic tumor microenvironment in MSI-H tumors.

## 3. Discussion

Our proteomic study revealed a significantly higher distribution of S100A14 in serrated lesions compared to healthy tissues and conventional adenomas. These findings were validated in an independent IHC cohort. Although various studies have identified S100A14 as upregulated in serrated lesions through proteomics and transcriptomics, none have confirmed this increase using orthogonal methods or investigated its role in serrated carcinogenesis [15,17,18].

S100A14 belongs to the S100 protein family, which plays multifunctional roles both intracellularly and extracellularly in cell growth and differentiation, Ca^2+^ homeostasis, and protein phosphorylation regulation. In our proteomic analysis, two other S100 family members, S100A16 (which physically interacts with S100A14) and S100P (known for its overexpression in SSL), were found to be more abundant in SSL than in DH, further reinforcing our data [19,20]. The S100 family has been implicated in tumorigenesis through the regulation of apoptosis, cell survival, migration, and immune evasion [21]. S100A14 is differentially expressed in various cancers, downregulated in gastrointestinal tumors, and upregulated in ovarian, breast, and lung cancers [22]. The heterogeneous expression profile of S100A14 across cancer types suggests a context-dependent, dual role for this protein, potentially acting as a tumor suppressor or promoter depending on the tissue microenvironment and molecular background.

In colorectal cancer, S100A14 has been found to be downregulated in tumors compared to normal tissues, and its low expression has been associated with poorer patient prognosis [23,24,25]. S100A14 could have a tumor suppressor role linked to a positive functional interaction with p53 [26]. However, the results highlighted in our study and our meta-analyses of existing datasets showed increased S100A14 expression in tumor lesions associated with the serrated pathway compared to tumors arising from the conventional adenoma–carcinoma sequence.

In a multi-omic study conducted by Chen et al., which compared conventional adenomas with serrated lesions, single-cell transcriptomic analysis suggested that adenomas are derived from cells with an intestinal stem cell signature (expressing *LGR5*, *OLFM4*, *ASCL2*), whereas serrated lesions originate from differentiated absorptive cells, such as goblet cells (expressing *TFF3* and *MUC2*). This is consistent with our proteomic and IHC observations of S100A14 increase in SSL, especially in the upper crypt region, which corresponds to mature differentiated cells. Furthermore, S100A14 has been implicated in the differentiation process of gastric tumor cells [27] and, more recently, its ability to reduce stemness in colorectal cancer cell lines has been established [28]. These two independent functional observations partially supported our observations.

In a study by Chen et al., serrated lesions were associated with a cytotoxic microenvironment enriched in CD8^+^ T cells. The same study identified *S100A14* as one of the top hits overexpressed in SSL compared to adenomas and normal tissues. Additionally, Zhou [29] and colleagues reported an enrichment of CD8^+^ CD103^+^ tumor-resident memory T cells (TRM) in SSL and MSS BRAF^V600E^ colorectal tumors. CRC with CD103^+^ TRM infiltration have been reported to predict the response to PD-L1 blocking therapy in several studies [30,31]. Taken together, given the upregulation of S100A14 in serrated lesions, the results presented in our study also support that S100A14 may participate in modulating the cytotoxic microenvironment of SSL.

Recently, Min et al. demonstrated that S100A14 regulates the immune invasion of colorectal cells by inhibiting PD-L1 expression through direct interaction with STAT3, leading to its proteasomal degradation [28]. This also suggests that the increased expression of S100A14 in SSL might prevent tumor immune evasion in a microenvironment rich in CD8^+^ T and NK cells. Furthermore, a recent study investigated the role of S100A14 in gastric cancer treatment outcomes by evaluating the combination of sintilimab (an anti-PD1 monoclonal antibody) and SOX therapy [32]. They demonstrated that S100A14 is located at the apical pole of tumor cells, where it interacts with lactotransferrin (LTF) in resident tumor macrophages, which is associated with a favorable response to treatment, suggesting that S100A14 may play a crucial role in mediating communication between tumor cells and the immune microenvironment.

Through the analysis of single-cell transcriptomic data generated by K. Pelka’s team, comparing MSI-H and MSS colorectal tumors, we identified an increased *S100A14* expression by tumor cells in MSI-H tumors compared to MSS tumors—a trend that we could also confirm by IHC in an independent set of patients. Moreover, we observed a positive correlation between malignant *S100A14* expression and the relative percentage of CD8^+^ cytotoxic T cells within the tumor, particularly CD8^+^ CXCL13^+^ cells. In a pan-tumor meta-analysis investigating T-cell mechanisms in sensitization to checkpoint inhibitors (CPI), intra-tumoral infiltration of CD8^+^ CXCL13^+^ cells has been described as a favorable indicator of response to immunotherapy. In addition, CXCL13 expression has been correlated with clonally reactive T lymphocytes that target tumor neoantigens. Consequently, CD8^+^ CXCL13^+^ cells have been proposed as predictive biomarkers to stratify patients prone to respond positively to CPI therapy [33]. This finding was further confirmed in a single-cell study examining the CPI response in CRC, where patients with an elevated baseline level of CD8^+^ CXCL13^+^ cells have been shown to present a complete response to treatment [34].

Up to 82% of serrated colorectal tumors are MSI-H [7] which is characterized by mucinous features and a high infiltration of T lymphocytes, as in SSL [13]. This suggests a potential role for S100A14 in preventing immune evasion in the cytotoxic microenvironment of SSL and MSI-H tumors. Moreover, given the better outcomes of MSI-H tumors and that S100A14 regulates PD-L1 expression, it would be interesting to explore its potential as a prognostic biomarker for immunotherapy responses based on PD-L1 targeting [35].

Our study presents some limitations linked to the low number of lesions included in the shotgun proteomic experiment, which is mitigated by the confirmations obtained using other technologies and other independent datasets generated using IHC, transcriptomic microarray, and single-cell public data.

In summary, our study highlights differential S100A14 abundance and expression in lesions known to originate from the conventional adenoma–carcinoma sequence from those resulting from the serrated transformation sequence, including sessile serrated lesions up to colorectal cancer. We hypothesize that S100A14 may play a role in shaping the anti-tumor immune microenvironment characteristic of SSL and MSI-H colorectal cancers, potentially serving as both a therapeutic target and a prognostic biomarker for predicting immunotherapy response. To further explore its clinical relevance, evaluating S100A14 expression in immunotherapy-treated patients stratified by response would be of high interest. Moreover, spatial transcriptomic/proteomic analyses could provide high-resolution insights into the spatial relationships between S100A14-expressing tumor cells and the immune landscape, not only in terms of immune cell composition but also their phenotypes within the tumor microenvironment. Overall, further investigations are needed to decipher the role of S100A14 in serrated neoplasia and its contribution to MSI-H CRC development.

## 4. Materials and Methods

### 4.1. Patient Inclusions and FFPE Tissue Samples

Formalin-fixed paraffin-embedded (FFPE) tissue blocks were obtained from the Biobank University Hospital of Liège. The patients and tissues included in this study were selected from our biobank archives (2008 to 2023).

Each lesion type was confirmed by a pathologist (NB and JS) using hematoxylin and eosin (H&E)-stained sections.

For the proteomic analysis, we included 20 DH, 15 SSL, 25 low-grade adenomas (LGA), and 20 high-grade adenomas tissues (HGA) (Supplementary Table S1). The tissues used for immunohistochemical confirmation were also obtained from other patients: 9 DH, 27 hyperplastic polyps (HP), 28 SSL (without dysplasia), 9 LGA, and 10 HGA (Supplementary Table S2). Colorectal cancer tissue samples used for IHC confirmations were selected based on microsatellite instability and *BRAF* mutation status determined by routine Next-Generation sequencing (NGS) (SOPHiA Solid Tumor Solution, SOPHiA GENETICS, Rolle, Suisse). The Lynch syndrome cases included were identified according to diagnostic guidelines based on genetic data [36]. The CRC cohort comprised 9 cases of CRC BRAF^WT^ MSS, 9 BRAF^V600E^ MSS, 10 BRAF^V600E^ MSI-H, and 8 Lynch syndrome cases (Supplementary Table S3).

### 4.2. Shotgun Proteomic Experiments

#### 4.2.1. Sample Processing

Each FFPE patient sample (6 µm tissue section) was macro-dissected to isolate the region containing the lesion of interest or healthy margins for normal tissues. For each lesion type, tissues from different patients were grouped and pooled to generate representative and biologically equivalent samples. Specifically, three separate pools were created for SSL, LGA, and HGA, and four pools for DH tissues, each comprising samples from multiple patients of the same lesion type. Each pool contained approximately 300 mm^3^ of tissue, based on the quantification of tissue slices macrodissected. Protein extraction and digestion from each pool were performed using the FFPE-FASP™ Protein Digestion Kit (Expedeon, Cambridge, UK) as previously described [37]. Briefly, each pool was weighed before and after deparaffinization to adjust the FASP-FFPE buffer volume accordingly (2.5 mg of dry material resuspended in 50 µL of UPX buffer). The protein concentration of each sample was determined using the RCDC Protein Assay Kit (Bio-Rad, Hercules, CA, USA) before digestion. An equivalent quantity of peptides (4.2 µg) per pool was desalted using C18 resin pipette tips (Zip Tip, Millipore Corp, Billerica, MA, USA), and the eluted fractions were pooled together and dried at RT using a speed vacuum. The pellets were stored at −20 °C prior to injection on a 2D-nanoUPLC system, with 3.0 µg of the digested protein content resuspended in 9 μL of 100 mM ammonium formate solution adjusted to pH 10.

The samples were spiked with MassPREP™ Digestion Standard Mixture (MPDS mix) (Waters Corp., Milford, CT, USA) as external standard, containing ENO1, P00924; GPB, P00489; ADH, P00330; and BSA, P02769 digests of known relative concentrations. The corresponding quantity of ADH spiked per injected sample was 150 fmol.

#### 4.2.2. LC-MSMS Analysis

The 13 peptide desalted mixtures were injected on a 2D-nanoAquity UPLC (Waters, Corp., Milford, CT, USA) coupled online with a Q Exactive™ Plus Hybrid Quadrupole-Orbitrap™ mass spectrometer (Thermo Fisher Scientific, Waltham, MA, USA) operated in positive ion mode. All parameters for peptide separation, MS, and MSMS data acquisition are detailed in [38]. In brief, peptide separation was performed using 2-dimension separation on hydrophobic columns at pH 10 and 3, with a 3-step elution and a useful 140 min. elution gradient. The Xcalibur program (ThermoFisher Scientific, Waltham, MA, USA) was used to record raw data for MS and subsequent data-dependent MS/MS scans of the 12 most intense ions. The MS spectrum acquisition ranged from 400 to 1750 *m*/*z* with resolution (at *m*/*z* 200) = 70,000, with an automatic gain control target of 1 × 10^6^, and a 200 ms maximum injection time. The MSMS spectra acquisition isolation window was 2.0 *m*/*z,* with a stepped normalized collision energy at 25 with a resolution of *m*/*z* 200 of 17,500, an AGC target at 1 × 10^5^, and a maximum injection time of 50 ms (1% underfill ratio).

#### 4.2.3. Protein Identifications and Quantifications

Protein identification and quantification were performed using MaxQuant vs. 1.5.5.1 [39] with protein searches performed on the Uniprot Human database (20,199 reviewed entries, released in January 2015) enriched with the sequences of the MPDS mix proteins and pig trypsin. Search parameters were trypsin as digestion enzyme, maximal number of miscleavages equal to 2, minimal peptide length at 7 amino acids, carbamidomethylation of cysteines as a fixed modification, methionine oxidation and N-acetylation as variable modifications, minimal number of peptides per protein equal to 2, minimal number of unique peptides per protein equal to 1, a precursor mass tolerance of 4.5 ppm, fragment mass tolerance of 0.5 Da, peptide and protein spectrum matches false discovery rates set at 0.01, and with match-between-run using a 0.7 min. time window. Data normalization and quantification were performed using the MaxQuant label-free quantification (LFQ) algorithm.

### 4.3. Immunohistochemistry (IHC) Experiments

#### 4.3.1. IHC Sample Preparation

FFPE tissue sections were prepared as in [37]. Tissue sections were incubated for 1h at RT with anti-S100A14 polyclonal antibody (dilution 1:500, Sigma-Aldrich (Darmstadt, Germany) Cat# HPA027613, RRID:AB_10602298) or anti-AKR1B10 polyclonal (dilution 1:300, Sigma-Aldrich Cat# HPA020280, RRID:AB_10602228) as primary antibodies.

#### 4.3.2. IHC Characterization

IHC staining intensity scores were established by two scorers blinded to the clinical and pathological classification (PA and CS). A semi-quantitative scale based on the average staining intensity of the whole tissue section, focusing on cytoplasmic epithelial staining, was used. The scoring scale used ranges from 0 (no staining), 1 (weak staining), 2 (intermediate staining), 3 (strong staining), and 4 (very strong staining). Any discrepancies in scoring were resolved until final agreement. The crypts were subdivided into three regions: lower crypt, middle crypt, and upper crypt, corresponding to different cell populations (stem cell-rich, transit-amplifying cells, and fully differentiated epithelial cells, respectively). To assess the distribution of AKR1B10 IHC staining, the presence of positive staining was evaluated in two epithelial compartments: the ‘upper third’, corresponding to differentiated epithelial cells, and the ‘upper two-thirds’, corresponding to immature and stem cells rich zones.

The average staining intensity of the tumor cells score was established within tumors due to architectural heterogeneity.

### 4.4. Gene Expression Analysis

Gene expression analyses were performed using two datasets: Gene Expression Omnibus (GEO) databases (accession numbers GSE75317 and GSE24550) containing expression data of colorectal cancer tissues. *S100A14* expression levels in different lesion types were compared using the GEO2R tool.

### 4.5. Single-Cell Data Analysis

Single-cell RNA-seq data were processed using the Seurat package (v5.1.0) (RRID:SCR_016341) in R environment (v4.4.1). Count matrices were downloaded from the Human Colon Cancer Atlas (c295) on Single-Cell Portal (https://singlecell.broadinstitute.org/single_cell/study/SCP1162/human-colon-cancer-atlas-c295 (accessed on 27 March 2024)) (GSE178341) and imported with the Read10X function. After quality control, cells with mitochondrial RNA over 5% were excluded from analysis. Data normalization (NormalizeData), variable feature selection, scaling (ScaleData), regressing-out mitochondrial RNA percentages, and total counts were performed. Dimensionality reduction and UMAP embedding were performed using RunPCA and RunUMAP functions, respectively. Metadata and cell annotation files were imported from the original analyses and were integrated into the Seurat object using AddMetaData.

### 4.6. Statistics

Proteomic differential analyses were performed using the Perseus software vs1.6.10.45 (RRID:SCR_015753), using MaxQuant (RRID:SCR_014485) output (combined file data protein table) [40]. Data were normalized after Log2 transformation. Reverse identifications were filtered out before category annotations corresponding to the 4 groups of sample pools analyzed (LGA, HGA, SSL, and N (normal tissues of DH)). The proteins of interest were Uniprot IDs showing significant differential distributions established using the ANOVA test or Welch tests for each comparison assessed and using a minimum of 50% of valid values in at least one group as inclusion filter criteria in LGA vs. N, HGA vs. N, SSL vs. N, and SSL vs. (LGA or HGA), providing an informative ranking of the proteins of interest according to their *p*-value and fold changes between groups. The minimum threshold of significance was set at *p* < 0.05.

S100A14 or AKR1B10 IHC scores and gene expression group comparison analyses were performed using the Kruskal–Wallis test, with post hoc multiple comparisons using Dunn’s test. For AKR1B10 distribution staining, the chi-square test was performed. Statistical tests and graphical illustrations were performed using GraphPad Prism v.9.5.0 (RRID:SCR_002798).

In single-cell analysis, Pearson correlation tests were used to assess the relationship between the average expression levels of *S100A14* in the epithelial tumor cell population of each donor and the proportion of immune cell populations in the corresponding tumor.

## Data Availability

Mass spectrometry proteomic data were deposited in the ProteomeXchange Consortium [41] via the PRIDE partner repository: dataset identifier PXD056781 [42].

## Supplementary Materials

The following supporting information can be downloaded at: https://www.mdpi.com/article/10.3390/ijms26157401/s1.

## Abbreviations

The following abbreviations are used in this manuscript:

ADH	Alcohol Dehydrogenase
AGC	Automatic Gain Control
AKR1B10	Aldo-Keto Reductase Family 1 Member B10
BRAF	v-Raf murine sarcoma viral oncogene homolog B
CCL5	Chemokine (C-C motif) Ligand 5
CPI	Checkpoint Inhibitor
CRC	Colorectal Cancer
CXCL13	Chemokine (C-X-C motif) Ligand 13
DH	Diverticulitis Healthy
ENO1	Enolase 1
FASP	Filter-Aided Sample Preparation
FFPE	Formalin-Fixed Paraffin-Embedded
GEO	Gene Expression Omnibus
GPB	Glycogen Phosphorylase B
H&E	Hematoxylin and Eosin
HGA	High-Grade Adenoma
HP	Hyperplastic Polyp
IHC	Immunohistochemistry
IL7R	Interleukin 7 Receptor
LC-MS/MS	Liquid Chromatography–Tandem Mass Spectrometry
LGA	Low-Grade Adenoma
LTF	Lactotransferrin
LGR5	Leucine-Rich Repeat Containing G Protein-Coupled Receptor 5
MMR	Mismatch Repair
MMRd	Mismatch Repair Deficient
MMRp	Mismatch Repair Proficient
MPDS	MassPREP Digestion Standard
MSI	Microsatellite Instability
MSI-L	Microsatellite Instability Low
MSI-H	Microsatellite Instability High
MSS	Microsatellite Stable
MVHP	Microvesicular Hyperplastic Polyp
MUC2	Mucin 2
MUC5A	Mucin 5AC
NGS	Next-Generation Sequencing
OLFM4	Olfactomedin 4
OR	Odds Ratio
p53	Tumor protein p53
PD-L1	Programmed Death-Ligand 1
PRIDE	PRoteomics IDEntifications Database
RCDC	Reducing Agent and Detergent Compatible
RRID	Research Resource Identifier
S100A14	S100 Calcium Binding Protein A14
S100A16	S100 Calcium Binding Protein A16
S100P	S100 Calcium Binding Protein P
SCP	Single Cell Portal
SSL	Sessile Serrated Lesion
STAT3	Signal Transducer and Activator of Transcription 3
TFF3	Trefoil Factor 3
TRM	Tissue-Resident Memory T cell
TSA	Traditional Serrated Adenoma
UMAP	Uniform Manifold Approximation and Projection
UPLC	Ultra-Performance Liquid Chromatography

## References

[1] Akimoto N., Ugai T., Zhong R., Hamada T., Fujiyoshi K., Giannakis M., Wu K., Cao Y., Ng K., Ogino S. (2021). Rising incidence of early-onset colorectal cancer: A call for action. Physiol. Behav..

[2] Müller M.F., Ibrahim A.E.K., Arends M.J. (2016). Molecular pathological classification of colorectal cancer. Virchows Arch..

[3] Okamoto K., Kitamura S., Kimura T., Nakagawa T., Sogabe M., Miyamoto H., Muguruma N., Takayama T. (2017). Clinicopathological characteristics of serrated polyps as precursors to colorectal cancer: Current status and management. J. Gastroenterol. Hepatol..

[4] Mezzapesa M., Losurdo G., Celiberto F., Rizzi S., D’amati A., Piscitelli D., Ierardi E., Di Leo A. (2022). Serrated Colorectal Lesions: An Up-to-Date Review from Histological Pattern to Molecular Pathogenesis. Int. J. Mol. Sci..

[5] Phipps A.I., Limburg P.J., Baron J.A., Burnett-Hartman A.N., Weisenberger D.J., Laird P.W., Sinicrope F.A., Rosty C., Buchanan D.D., Potter J.D. (2015). Association between molecular subtypes of colorectal cancer and patient survival. Gastroenterology.

[6] Ahadi M., Sokolova A., Brown I., Chou A., Gill A.J. (2021). The 2019 World Health Organization Classification of appendiceal, colorectal and anal canal tumours: An update and critical assessment. Pathology.

[7] Satorres C., García-Campos M., Bustamante-Balén M. (2021). Molecular Features of the Serrated Pathway to Colorectal Cancer: Current Knowledge and Future Directions. Gut Liver.

[8] Li D., Doherty A.R., Raju M., Liu L., Lei N.Y., Amsden L.B., Lee J.K., Levin T.R., Corley D.A., Herrinton L.J. (2022). Risk stratification for colorectal cancer in individuals with subtypes of serrated polyps. Gut.

[9] Yang J.F., Tang S.J., Lash R.H., Wu R., Yang Q. (2015). Anatomic distribution of sessile serrated adenoma/polyp with and without cytologic dysplasia. Arch. Pathol. Lab. Med..

[10] Haque T., Greene K.G., Crockett S.D. (2014). Serrated neoplasia of the colon: What do we really know?. Curr. Gastroenterol. Rep..

[11] Crockett S.D., Nagtegaal I.D. (2019). Terminology, Molecular Features, Epidemiology, and Management of Serrated Colorectal Neoplasia. Gastroenterology.

[12] Murcia O., Juárez M., Rodríguez-Soler M., Hernández-Illán E., Giner-Calabuig M., Alustiza M., Egoavil C., Castillejo A., Alenda C., Barberá V. (2018). Colorectal cancer molecular classification using BRAF, KRAS, microsatellite instability and CIMP status: Prognostic implications and response to chemotherapy. PLoS ONE.

[13] De’angelis G.L., Bottarelli L., Azzoni C., De’angelis N., Leandro G., Di Mario F., Gaiani F., Negri F. (2018). Microsatellite instability in colorectal cancer. Acta Biomed..

[14] Zhao P., Li L., Jiang X., Li Q. (2019). Mismatch repair deficiency/microsatellite instability-high as a predictor for anti-PD-1/PD-L1 immunotherapy efficacy. J. Hematol. Oncol..

[15] Sohier P., Sanson R., Leduc M., Audebourg A., Broussard C., Salnot V., Just P.A., Pasmant E., Mayeux P., Guillonneau F. (2020). Proteome analysis of formalin-fixed paraffin-embedded colorectal adenomas reveals the heterogeneous nature of traditional serrated adenomas compared to other colorectal adenomas. J. Pathol..

[16] Pelka K., Hofree M., Chen J.H., Sarkizova S., Pirl J.D., Jorgji V., Bejnood A., Dionne D., Ge W.H., Xu K.H. (2021). Spatially organized multicellular immune hubs in human colorectal cancer. Cell.

[17] Rickelt S., Condon C., Mana M., Whittaker C., Pfirschke C., Roper J., Patil D.T., Brown I., Mattia A.R., Zukerberg L. (2020). Agrin in the Muscularis mucosa serves as a biomarker distinguishing hyperplastic polyps from sessile serrated lesions. Clin. Cancer Res..

[18] Chen B., Scurrah C.R., McKinley E.T., Simmons A.J., Ramirez-Solano M.A., Zhu X., Markham N.O., Heiser C.N., Vega P.N., Rolong A. (2021). Differential pre-malignant programs and microenvironment chart distinct paths to malignancy in human colorectal polyps. Cell.

[19] Basnet S., Vallenari E.M., Maharjan U., Sharma S., Schreurs O., Sapkota D. (2023). An Update on S100A16 in Human Cancer. Biomolecules.

[20] Takahashi S., Okamoto K., Tanahashi T., Fujimoto S., Nakagawa T., Bando M., Ma B., Kawaguchi T., Fujino Y., Mitsui Y. (2021). S100P Expression via DNA Hypomethylation Promotes Cell Growth in the Sessile Serrated Adenoma/Polyp-Cancer Sequence. Digestion.

[21] Bresnick A.R., Weber D.J., Zimmer D.B. (2015). S100 proteins in cancer. Nat. Rev. Cancer.

[22] Basnet S., Sharma S., Costea D.E., Sapkota D. (2019). Expression profile and functional role of S100A14 in human cancer. Oncotarget.

[23] Hashida H., Coffey R.J. (2022). Significance of a calcium-binding protein S100A14 expression in colon cancer progression. J. Gastrointest. Oncol..

[24] Wang H.-Y., Zhang J.-Y., Cui J.-T., Tan X.-H., Li W.-M., Gu J., Lu Y.-Y. (2010). Expression status of S100A14 and S100A4 correlates with metastatic potential and clinical outcome in colorectal cancer after surgery. Oncol. Rep..

[25] Diamantopoulou A., Mantas D., Kostakis I.D., Agrogiannis G., Garoufalia Z., Kavantzas N., Kouraklis G. (2020). A clinicopathological analysis of S100A14 expression in colorectal cancer. In Vivo.

[26] Sapkota D., Costea D.E., Blø M., Bruland O., Lorens J.B., Vasstrand E.N., Ibrahim S.O. (2012). S100A14 inhibits proliferation of oral carcinoma derived cells through G1-arrest. Oral. Oncol..

[27] Zhu M., Wang H., Cui J., Li W., An G., Pan Y., Zhang Q., Xing R., Lu Y. (2017). Calcium-binding protein s100a14 induces differentiation and suppresses metastasis in gastric cancer. Cell Death Dis..

[28] Min H., Cho J., Sim J.Y., Boo H., Lee J., Lee S., Lee Y., Kim S.J., Kim K., Park I. (2022). S100A14: A novel negative regulator of cancer stemness and immune evasion by inhibiting STAT3-mediated programmed death-ligand 1 expression in colorectal cancer. Clin. Transl. Med..

[29] Zhou Y.-J., Lu X.-F., Chen H., Wang X.-Y., Cheng W., Zhang Q.-W., Chen J.-N., Wang X.-Y., Jin J.-Z., Yan F.-R. (2022). Single-cell transcriptomics reveals early molecular and immune alterations underlying the serrated neoplasia pathway toward colorectal cancer. Cell. Mol. Gastroenterol. Hepatol..

[30] Banchereau R., Chitre A.S., Scherl A., Wu T.D., Patil N.S., De Almeida P., Kadel E.E., Madireddi S., Au-Yeung A., Takahashi C. (2021). Intratumoral CD103^+^ CD8^+^ T cells predict response to PD-L1 blockade. J. Immunother. Cancer.

[31] Okla K., Farber D.L., Zou W. (2021). Tissue-resident memory T cells in tumor immunity and immunotherapy. J. Exp. Med..

[32] Che G., Yin J., Wang W., Luo Y., Chen Y., Yu X., Wang H., Liu X., Chen Z., Wang X. (2024). Circumventing drug resistance in gastric cancer: A spatial multi-omics exploration of chemo and immuno-therapeutic response dynamics. Drug Resist. Updat..

[33] Litchfield K., Reading J.L., Puttick C., Thakkar K., Abbosh C., Bentham R., Watkins T.B.K., Rosenthal R., Biswas D., Rowan A. (2021). Meta-analysis of tumor- and T cell-intrinsic mechanisms of sensitization to checkpoint inhibition. Cell.

[34] Chen Y., Wang D., Li Y., Qi L., Si W., Bo Y., Chen X., Ye Z., Fan H., Liu B. (2024). Spatiotemporal single-cell analysis decodes cellular dynamics underlying different responses to immunotherapy in colorectal cancer. Cancer Cell.

[35] Grasso C.S., Giannakis M., Wells D.K., Hamada T., Mu X.J., Quist M., Nowak J.A., Nishihara R., Qian Z.R., Inamura K. (2018). Genetic mechanisms of immune evasion in colorectal cancer. Cancer Discov..

[36] Hébrant A., Jouret-Mourin A., Froyen G., Van Der Meulen J., De Man M., Salgado R., Van Den Eynde M., D’haene N., Martens G., Van Cutsem E. (2019). Molecular test algorithms for digestive tumours. Belg. J. Med. Oncol..

[37] Quesada-Calvo F., Massot C., Bertrand V., Longuespée R., Blétard N., Somja J., Mazzucchelli G., Smargiasso N., Baiwir D., De Pauw-Gillet M.-C. (2017). OLFM4, KNG1 and Sec24C identified by proteomics and immunohistochemistry as potential markers of early colorectal cancer stages. Clin. Proteom..

[38] Pierre N., Salée C., Massot C., Blétard N., Mazzucchelli G., Smargiasso N., Morsa D., Baiwir D., De Pauw E., Reenaers C. (2020). Proteomics Highlights Common and Distinct Pathophysiological Processes Associated with Ileal and Colonic Ulcers in Crohn’s Disease. J. Crohn's Colitis.

[39] Tyanova S., Temu T., Cox J. (2016). The MaxQuant computational platform for mass spectrometry-based shotgun proteomics. Nat. Protoc..

[40] Tyanova S., Temu T., Sinitcyn P., Carlson A., Hein M.Y., Geiger T., Mann M., Cox J. (2016). The Perseus computational platform for comprehensive analysis of (prote)omics data. Nat. Methods.

[41] Deutsch E.W., Bandeira N., Perez-Riverol Y., Sharma V., Carver J.J., Mendoza L., Kundu D.J., Wang S., Bandla C., Kamatchinathan S. (2023). The ProteomeXchange consortium at 10 years: 2023 update. Nucleic Acids Res..

[42] Perez-Riverol Y., Bai J., Bandla C., García-Seisdedos D., Hewapathirana S., Kamatchinathan S., Kundu D.J., Prakash A., Frericks-Zipper A., Eisenacher M. (2022). The PRIDE database resources in 2022: A hub for mass spectrometry-based proteomics evidences. Nucleic Acids Res..

