# Supplementary material for: S100A14 as a Potential Biomarker of the Colorectal Serrated Neoplasia Pathway
Source: Int J Mol Sci. 2025 Jul 31;26(15):7401. doi: 10.3390/ijms26157401 (PMC12347601; doi:10.3390/ijms26157401)
Supplement: Supplementary file 1 [file ijms-26-07401-s001.zip › Supplementary table S1 S2 S3 and figure S1-layout1.pdf]

Supplementary Table S1, S2, S3 and figure S1

Supplementary Table S1. Patients' characteristics of proteomic cohort.

	Diverticular disease	SSL	Adenoma low grade	Adenoma high grade
Patient, [gender: m/f], n	20 [11/9]	15 [11/4]	25 [9/16]	20 [14/6]
Age, median [min-max], years	63 [51-73]	66 [47-77]	66 [49-93]	69 [41-89]
Lesion location, n				
Rectum	2	2	3	4
Sigmoid	16	2	7	7
Colon	2	7	9	9
Caecum	0	4	6	0

Supplementary Table S2. Patients' characteristics of IHC validation cohort.

	Diverticular disease	Hyperplastic	SSL	Adenoma low grade	Adenoma high grade
Patient, [gender: m/f], n	9 [5/4]	27 [11/16]	28 [15/13]	9 [5/4]	10 [6/4]
Age, median [min-max], years	58.0 [44.0-81.3]	59.2 [35.5-74.4]	62.6 [34.9-75.7]	64.1 [53.9-71.2]	69.6 [49.3-92.9]
Smoking / alcohol consumption, n					
Active	2/0	11/14	8/10	2/3	3/3
Former	1/0	0/0	5/0	0/0	0/0
None	5/8	15/12	8/11	1/1	4/5
NR (not recorded)	1/1	1/1	7/7	6/5	3/2
Lesion location, n					
Rectum	0	7	2	0	4
Sigmoid	8	12	5	2	3
Left colon	1	0	4	3	0
Transverse colon	0	1	3	0	0
Right colon	0	4	7	2	0
Caecum	0	2	7	1	1
NR	0	0	0	1	2
BRAFV600E, n					
Negative	10	7	4	9	9
Positive	0	19	24	1	1

NR: not recorded

Supplementary Table S3. Patients' characteristics of CRC cohort.

	BRAF WT MSS	BRAF V600E MSS	BRAF V600E MSI-H	LYNCH
Patient, [gender: m/f], n	9 [4/5]	9 [3/6]	10 [1/9]	8 [6/2]
Age, median [min-max], years	75.6 [44.9-84.8]	60.0 [27.4-78.2]	74.7 [63.3-84.2]	48.3 [32.6-65.1]
Smoking / alcohol consumption, n				
Active	2/5	0/4	4/3	3/7
Former	1/0	0/0	2/0	1/0
None	5/3	7/4	4/7	4/1
NR (not recorded)	1/1	2/1	0/0	0/0
Lesion location, n				
Rectum	1	0	0	1
Sigmoid	6	2	1	1
Left colon	0	0	0	2
Transverse colon	1	3	0	3
Right colon	0	3	6	1
Caecum	1	1	3	0
NR	0	0	0	0
pTNM, n				
T1	0	0	1	2
T2	0	0	2	0
T3	3	2	4	4
T4	1	3	3	1
Nx	0	0	0	0
N0	1	0	9	4
N1	2	2	0	2
N2	1	3	1	1
Mx	4	2	10	7
M0	0	0	0	0
M1	0	3	0	0
NR	5	4	0	1

NR: not recorded

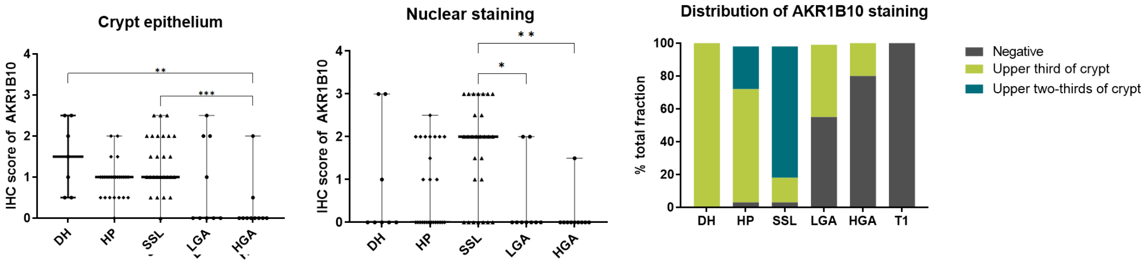

Supplementary Figure S1. AKR1B10 IHC scoring in crypt epithelium, nuclear and frequency distribution according to the staining patterns (upper third or upper two-third of the crypts). (Chi-square p-value = 0.0004 considering only SSL and HP).*p < 0.05, ** p<0.001, *** p<0.001 by kruskal-Wallis test using Dunn's multiple comparaisons post hoc test.
